# Global population genetics and diversity in the TAS2R bitter taste receptor family

**DOI:** 10.3389/fgene.2022.952299

**Published:** 2022-10-11

**Authors:** Stephen P. Wooding, Vicente A. Ramirez

**Affiliations:** ^1^ Department of Anthropology, University of California, Merced, Merced, CA, United States; ^2^ Department of Public Health, University of California, Merced, Merced, CA, United States

**Keywords:** taste, genetics, perception, selection, evolution

## Abstract

Bitter taste receptors (TAS2Rs) are noted for their role in perception, and mounting evidence suggests that they mediate responses to compounds entering airways, gut, and other tissues. The importance of these roles suggests that *TAS2R*s have been under pressure from natural selection. To determine the extent of variation in *TAS2R*s on a global scale and its implications for human evolution and behavior, we analyzed patterns of diversity in the complete 25 gene repertoire of human *TAS2R*s in ∼2,500 subjects representing worldwide populations. Across the *TAS2R* family as a whole, we observed 721 single nucleotide polymorphisms (SNPs) including 494 nonsynonymous SNPs along with 40 indels and gained and lost start and stop codons. In addition, computational predictions identified 169 variants particularly likely to affect receptor function, making them candidate sources of phenotypic variation. Diversity levels ranged widely among loci, with the number of segregating sites ranging from 17 to 41 with a mean of 32 among genes and per nucleotide heterozygosity (π) ranging from 0.02% to 0.36% with a mean of 0.12%. *F*_ST_ ranged from 0.01 to 0.26 with a mean of 0.13, pointing to modest differentiation among populations. Comparisons of observed π and *F*_ST_ values with their genome wide distributions revealed that most fell between the 5th and 95th percentiles and were thus consistent with expectations. Further, tests for natural selection using Tajima’s *D* statistic revealed only two loci departing from expectations given *D*’s genome wide distribution. These patterns are consistent with an overall relaxation of selective pressure on *TAS2R*s in the course of recent human evolution.

## Introduction

Bitter taste perception plays key roles in vertebrate biology and fitness. By enabling the detection of toxic defense compounds found in plants, it allows herbivores to monitor intake, preventing overexposure (Crozier et al., 2006; Reed and Knaapila, 2010). The protective role of bitter taste appears to be reduced in modern humans, whose diets consist mainly of domestic crops bred to be nontoxic. Nonetheless, bitter perception continues to shape behavior and health. For instance, many substances perceived as bitter are nontoxic, and avoidance is unnecessary (Nissim et al., 2017; Glendinning, 2021). Such is the case with cruciferous vegetables. They are healthy but often contain harmless bitter compounds that discourage consumption. Complexity is added by the fact that the toxicity of bitter substances is not binary but continuous, ranging from harmless to highly dangerous (Nissim et al., 2017). Bitter perception also plays less obvious roles. Notably, the molecular mechanisms underlying bitter taste are found in chemosensory cells in the gut and airways, where they mediate cellular responses to ingested and inhaled substances (Deshpande et al., 2010; Calvo and Egan, 2015). Further, variation in bitter taste sensitivity is associated with ingestion related health measures such as tobacco use and body mass index (BMI), as well as extraoral traits including glucose homeostasis and resistance to respiratory infection (Dotson et al., 2008; Lee et al., 2012).

Bitter taste responses are initiated by receptors in the TAS2R family, a series of G protein-coupled receptors (GPCRs) allied to other sensory receptors including opsins (OPNs) and olfactory receptors (ORs) (Chaudhari and Roper, 2010). TAS2Rs are expressed on the surface of taste receptor cells (TRCs) in taste buds, where they are exposed to ingested substances. When exposed to agonists they trigger a transduction cascade that depolarizes the cell and generates a neural signal. Humans express 25 TAS2R isoforms, each of which is receptive to a different range of compounds. Scores of TAS2R agonists have been identified experimentally, and it is now known that most TAS2Rs are capable of responding to numerous compounds, indicating that the full range of compounds capable of eliciting bitter tastes is vast (Meyerhof et al., 2010; Dagan-Wiener et al., 2019). The extent to which TAS2R expression and response patterns in extraoral tissues mirror those in TRCs is not well investigated. However a number of TAS2Rs are expressed in chemosensory cells the airway and gut, suggesting those tissues too are responsive to diverse agonists (Martens et al., 2021).

The genes encoding TAS2R receptors harbor extensive polymorphism, including numerous nonsynonymous variants (Kim et al., 2005). Nonsynonymous variation is a common source of phenotypic variation, and its presence in *TAS2R*s results in variation in perception. The most thoroughly documented example of the relationship is between variation at *TAS2R38* and perception of isothiocyanates (ITCs), a class of compounds that imparts bitterness to many vegetables (Kim et al., 2003; Wooding et al., 2010). *TAS2R38* harbors nonsynonymous variants that alter its responses to ITCs, and associations between *TAS2R38* genotypes and taste sensitivity to goitrin, a bitter yet harmless ITC found in many vegetables, are strong (Bufe et al., 2005; Wooding et al., 2010). Variation in *TAS2R38* also associates with preferences for downstream health variables relating to vegetable consumption such as adiposity (Tepper et al., 2009; Duffy et al., 2010). Thus, *TAS2R38* mediates a path from perception to health.

Findings similar to those surrounding *TAS2R38* continue to emerge at other loci. For instance, *TAS2R44* harbors numerous nonsynonymous variants shaping its responses to the bitter off-tastes of artificial sweeteners such as saccharin, which associate with perception (Roudnitzky et al., 2011). Likewise, while some variants of *TAS2R44* associate with sensitivity to the bitterness of saccharin, others do not, and whether they associate with behaviors such as consumption of low calorie drinks is not known. Additional associations between variation in *TAS2R*s, functional variation, and phenotypic variation have also emerged (Pronin et al., 2007; Intelmann et al., 2009; Roudnitzky et al., 2011; Hayes et al., 2015; Nolden et al., 2016). Given the large number of TAS2Rs in humans and their responses to a broad constellation of compounds, it is likely many more such relationships exist. An essential step forward in defining them will be to fully characterize of the extent of polymorphism in *TAS2R*s, its diversity and population structure, and its evolutionary origins.

In this study, we sought to determine the extent of genetic diversity in *TAS2R*s on a global scale and its implications for the evolution of bitter perception, functional variation, and similarities and differences among populations. To obtain a full portrait of variation in *TAS2R*s, we cataloged variation across the complete *TAS2R* family in humans by analyzing whole genome sequences in ∼2,500 worldwide subjects. We then used computational methods to predict the functional consequences of discovered variants and tested for signatures of natural selection to determine their influences during recent human evolution. Finally, we determined the distribution of *TAS2R* variation across human populations to shed light on the potential for phenotypic similarities and differences between them.

## Materials and methods

We examined genetic variation across the *TAS2R* family in data available from Phase 3 of the 1,000 Genomes Project (1,000GP) (The 1000 Genomes Project Consortium, 2015) (Table 1). The 1,000GP sample comprises 2,504 healthy, randomly selected subjects from 26 worldwide populations in five continental super populations. The complete *TAS2R* repertoire in humans includes 25 annotated functional loci and seven pseudogenes. However, because we focused on functional variation, we excluded the pseudogenes from analysis. Two *TAS2R*s known to have high frequency whole gene deletion alleles, *TAS2R43* and *TAS2R45*, were also excluded, because the presence of deletion alleles causes errors in genotype calls estimates of allele frequency (Roudnitzky et al., 2011). Thus, our final sample was composed of complete polymorphism data from 23 *TAS2R*s in 2,405 individuals.

**TABLE 1:** Sampled populations and super populations.

Super population	Population
Africa (*N* = 661)	African Caribbeans in Barbados (*N* = 96)
	Americans of African Ancestry in SW United States (*N* = 61)
	Esan in Nigeria (*N* = 99)
	Gambian in Western Divisions in the Gambia (*N* = 113)
	Luhya in Webuye, Kenya (*N* = 99)
	Mende in Sierra Leone (*N* = 85)
	Yoruba in Ibadan, Nigeria (*N* = 108)
Americas (*N* = 347)	Colombians from Medellin, Colombia (*N* = 94)
	Mexican Ancestry from Los Angeles, United States (*N* = 64)
	Peruvian from *Lima*, Peru (*N* = 85)
	Puerto Rican in Puerto Rico (*N* = 104)
East Asia (*N* = 504)	Chinese Dai in Xishuangbanna, China (*N* = 93)
	Han Chinese in Beijing, China (*N* = 103)
	Japanese in Tokyo, Japan (*N* = 104)
	Kinh in Ho Chi Minh City, Vietnam (*N* = 99)
	Southern Han Chinese (*N* = 105)
Europe (N = 503)	British in England and Scotland (*N* = 91)
	Finnish in Finland (*N* = 99)
	Iberian population in Spain (*N* = 107)
	Toscani in Italia (*N* = 107)
	Utah residents (CEPH) with European ancestry (*N* = 99)
South Asia (*N* = 489)	Bengali from Bangladesh (*N* = 86)
	Gujarati Indian from Houston, TX (*N* = 103)
	Indian Telegu from the United Kingdom (*N* = 102)
	Punjabi from Lahore, Pakistan (*N* = 96)
	Sri Lankan Tamil from the United Kingdom (*N* = 102)

We obtained the genomic coordinates of *TAS2R*s from the Ensembl hg19/GRCh37 human genome assembly, the reference genome for the 1,000GP (Table 2). Ensembl Gene ID and Transcript IDs were obtained using the Ensembl browser. The transcript IDs were used to extract variation data from the 1,000GP database in variant call format (vcf) using the Tabix software package (Li, 2011). Variants were then categorized as synonymous, nonsynonymous, premature stop, lost stop, lost start, and frameshift using annotations obtained from the Ensembl database using the Variant Effect Predictor (VEP) (Mclaren et al., 2016).

**TABLE 2:** *TAS2R* genes, coordinates, and sizes.

Gene	GRCh 37 coordinates	Transcript length	# Codons
*TAS2R1*	5:9629109–9630463	1,355	300
*TAS2R3*	7:141463897–141464997	1,101	317
*TAS2R4*	7:141478242–141479235	994	300
*TAS2R5*	7:141490017–141491166	1,150	300
*TAS2R7*	12:10954131–10955226	1,096	319
*TAS2R8*	12:10958650–10959892	1,243	310
*TAS2R9*	12:10961693–10962767	1,075	313
*TAS2R10*	12:10977916–10978957	1,042	308
*TAS2R13*	12:11060525–11062161	1,637	304
*TAS2R14*	12:11090005–11091862	1,858	318
*TAS2R16*	7:122634759–122635754	996	292
*TAS2R19*	12:11174218–11175219	1,002	300
*TAS2R20*	12:11149094–11150474	1,381	310
*TAS2R30*	12:11285557–11287243	1,687	320
*TAS2R31*	12:11182986–11184006	1,021	310
*TAS2R38*	7:141672431–141673573	1,143	334
*TAS2R39*	7:142880512–142881528	1,017	339
*TAS2R40*	7:142919130–142920162	1,033	324
*TAS2R41*	7:143174966–143175889	924	308
*TAS2R42*	12:11338599–11339543	945	315
*TAS2R43*	na:na-na	na	na
*TAS2R45*	na:na-na	na	na
*TAS2R46*	12:11213964–11214893	930	310
*TAS2R50*	12:11138512–11139511	1,000	300
*TAS2R60*	7:143140546–143141502	957	319

Genetic diversity was assessed at each locus using three measures, the number of segregating sites (*S*), nucleotide diversity (π) and population substructure (*F*_ST_). *S* was defined as the number of variable nucleotide positions in a sequence. π was defined as mean pairwise nucleotide difference between sequences normalized to sequence length. *F*_ST_ is the fraction of diversity accounted for by between-population differences. Here, we calculated *F*_ST_ using the weighted method of Weir and Cockerham (1984) These calculations were performed using VCFtools and the R packages PopGenome, heirfstat, pegas, adegnet, gdsfmt, and SNPRelate packages (Goudet, 2004; Paradis, 2010; Danecek et al., 2011; Jombart and Ahmed, 2011; Pfeifer et al., 2014).

We used two algorithms to predict the functional impact of non-synonymous variants, PolyPhen-2 and SIFT (Kumar et al., 2009; Adzhubei et al., 2010). PolyPhen-2 predicts the impact of amino acid substitutions based on the position of the changes in the three dimensional structure of the protein, conservation relative to homologous genes, and the biochemical characteristics of the substituted amino acids. PolyPhen-2 scores range from 0.0 to 1.0, and represent the probability that the substitution is damaging. PolyPhen-2 also provides qualitative ratings based on the probabilities, including benign (probability 0.000–0.446), possibly damaging (0.446–0.908), and probably damaging (0.908). SIFT predicts the impact of amino acid substitutions based on evolutionary conservation relative to homologous genes, and physiochemical similarity between the amino acids substituted. SIFT scores range from 0.0 to 1.0 and estimate the probability that the variant is deleterious or tolerated. SIFT scores < 0.05 are categorized as deleterious and scores > 0.05 are categorized as tolerated. To further examine the distribution of variation with respect to TAS2Rs’ molecular structure, which is also potentially indicative of variants’ functional effects, we aligned the amino acid sequences of the 23 putatively functional receptors using Clustal *W* and determined the abundance of variable sites across secondary structures and individual residues across the length of the molecule (Sievers et al., 2011).

Tests for natural selection were performed using Tajima’s *D* statistic, which compares the number of nucleotide differences between alleles with the number of variable sites, which are differentially affected by selective processes (Tajima, 1989). *D* tests are designed to detect selective effects in nonrecombining genomic regions but are applicable with elevated conservativeness to regions with recombination. Because TAS2Rs are small (∼1 kb–2 kb), recombination rates in them are nonzero but likely low. Therefore, the results of *D* tests were slightly conservative in our sample. These analyses were performed using the PopGenome R package.

A key assumption of selection tests using Tajima’s *D* is that population sizes have been constant. This assumption is violated in humans, whose populations have grown explosively over the last 60,000 years (Henn et al., 2012). Various strategies can be used control for this (Williamson et al., 2005). We took an empirical approach, using noncoding regions of the genome as a basis for comparisons. Roughly 98% of the human genome is thought to be noncoding. These regions do contain substantial functional sequence (Encode Project Consortium, 2012); however, the maximum estimated functional content in noncoding regions is < 15% and is likely less than half that (Graur, 2017). Therefore, to approximate the neutral distribution of *D* in our sample, we iteratively calculated it in adjacent 1 kb windows spanning the length of the 1,000GP genomes, excluding annotated functional elements and known unstable and repetitive regions such as telomeres. We then defined the probability of observed *D* values as their percentile position in the genome-wide distribution. We denoted *p*-values from these empirical tests *P*_E_-values to distinguish them from the *p*-values obtained in standard parametric tests. We used the same methodology to determine the *P*_E_ values of observed π and *F*_ST_ values. These analyses were performed using VCFTools and R (Danecek et al., 2011).

In addition to using *F*_ST_, we examined population structure using principal components analyses (PCA). To gain insight into the potential for population differences in functional variation we performed these for all sites, non-synonymous sites, and synonymous sites. These analyses were performed using the gdsfmt and SNPrelate R packages and visualized using the ggplot2 library (Zheng et al., 2012; Wickham, 2016).

## Results

Across our sample as a whole, we observed 721 single nucleotide polymorphisms, 9 short insertion-deletion polymorphisms, and 8 large structural variants (Tables 3, 4). The number of coding region variants ranged from 15 in *TAS2R13* to 40 in *TAS2R31*. The minor allele frequency (MAF) of variants across ranged from 0.0002 to 0.943 with an average of 0.033. However, their distribution was skewed strongly downward. The majority of sites (*n* = 447) were observed just once in the sample, and thus were singletons. Eighty percent of sites had allele frequencies below 0.004 and 95% had allele frequencies below 0.34. Alleles with intermediate frequencies, between 0.25 and 0.75, accounted for 4.5% of sites.

**TABLE 3:** Summary of variation in *TAS2R*s. The four main columns are segregating sites (*S*), nucleotide diversity (π), Tajima’s *D* statistic (*D*), and population differentiation (*F*_ST_). The percentile (% ile) of each value relative to the genome wide empirical distribution is given for π, *D*, and *F*_ST_. Separate values for nonsynonymous (*n*) and synonymous (s) sites are given for *S* and *F*_ST_.

Gene	*S*	(n, s)	π (%)	(%ile)	*D*	(%ile)	*F*_ST_	(%ile)	(n, s)
*TAS2R1*	27	(21, 6)	0.062	(42.1)	−2.02	(22.3)	0.18	(92.7)	(0.16, 0.01)
*TAS2R3*	26	(17, 9)	0.104	(65.2)	−1.49	(68.3)	0.10	(68.9)	(0.00, 0.14)
*TAS2R4*	30	(23, 7)	0.160	(84.1)	−1.22	(82.3)	0.14	(86.5)	(0.15, 0.02)
*TAS2R5*	33	(25, 8)	0.108	(67.3)	−1.90	(32.9)	0.13	(81.5)	(0.13, 0.04)
*TAS2R7*	40	(31, 9)	0.033	(22.5)	−2.20	(7.5)	0.05	(30.4)	(0.04, 0.01)
*TAS2R8*	32	(26, 6)	0.072	(48.4)	−1.98	(26.1)	0.21	(96.0)	(0.26, 0.20)
*TAS2R9*	31	(27, 4)	0.058	(39.1)	−1.91	(32.1)	0.17	(91.4)	(0.17, 0.00)
*TAS2R10*	32	(27, 5)	0.030	(20.8)	−2.17	(9.5)	0.14	(85.9)	(0.11, 0.20)
*TAS2R13*	17	(16, 1)	0.065	(44.0)	−1.66	(55.1)	0.23	(96.8)	(0.23, 0.00)
*TAS2R14*	30	(21, 9)	0.077	(51.6)	−1.82	(41.0)	0.14	(85.9)	(0.16, 0.15)
*TAS2R16*	36	(20, 16)	0.115	(70.5)	−1.73	(49.1)	0.22	(96.1)	(0.18, 0.25)
*TAS2R19*	31	(25, 6)	0.160	(84.1)	−1.25	(81.0)	0.10	(71.5)	(0.07, 0.13)
*TAS2R20*	36	(27, 9)	0.358	(98.6)	−0.25	(98.0)	0.26	(98.1)	(0.25, 0.30)
*TAS2R30*	33	(22, 11)	0.144	(80.0)	−1.25	(80.8)	0.07	(48.2)	(0.07, 0.07)
*TAS2R31*	41	(36, 5)	0.232	(93.8)	−1.17	(84.2)	0.06	(45.9)	(0.08, 0.00)
*TAS2R38*	32	(22, 10)	0.136	(77.7)	−1.32	(77.9)	0.08	(57.9)	(0.08, 0.00)
*TAS2R39*	32	(23, 9)	0.005	(3.5)	−2.26	(5.5)	0.01	(2.6)	(0.01, 0.00)
*TAS2R40*	28	(16, 12)	0.020	(14.9)	−2.13	(12.2)	0.03	(14.0)	(0.03, 0.00)
*TAS2R41*	31	(18, 13)	0.078	(51.7)	−1.81	(42.2)	0.09	(65.6)	(0.09, 0.09)
*TAS2R42*	36	(24, 12)	0.335	(98.2)	−-0.47	(96.8)	0.22	(96.5)	(0.20, 0.30)
*TAS2R43*	na	(na, na)	na	(na)	na	(na)	na	(na)	(na, na)
*TAS2R45*	na	(na, na)	na	(na)	na	(na)	na	(na)	(na, na)
*TAS2R46*	32	(25, 7)	0.119	(71.8)	−1.58	(61.9)	0.05	(38.6)	(0.04, 0.10)
*TAS2R50*	28	(16, 12)	0.123	(73.4)	−1.46	(70.0)	0.23	(97.1)	(0.29, 0.19)
*TAS2R60*	34	(23, 11)	0.051	(33.3)	−2.02	(22.1)	0.10	(72.9)	(0.03, 0.12)

**TABLE 4:** PHI sites with minor allele frequency (MAF) > 0.001. Variant type abbreviations are nonsynonymous (Ns), stop gained (Sg), inframe change (If), frameshift change (Fs). Ref.and Alt. Indicate reference and alternate codons and amino acids at the given position.

Gene	rsid	Var Type	Ref.Codon	Alt.Codon	Ref. AA	Alt. AA	MAF
*TAS2R42*	rs1669412	Ns	cGa	cAa	R	Q	0.2200
*TAS2R46*	rs2708381	Sg	tGg	tAg	W	Stop	0.2119
*TAS2R60*	rs35195910	If	gTCTtc	gtc	VF	V	0.0321
*TAS2R7*	rs77050900	Ns	aTt	aCt	I	T	0.0194
*TAS2R31*	rs139069360	Ns	tgG	tgT	W	C	0.0144
*TAS2R46*	rs201847607	Fs	Tgg	gg	W	X	0.0136
*TAS2R9*	rs113883583	Ns	gGg	gAg	G	E	0.0122
*TAS2R31*	rs116926686	Ns	ttA	ttT	L	F	0.0102
*TAS2R10*	rs117936881	Ns	Tgg	Cgg	W	R	0.0082
*TAS2R8*	rs200711805	Fs	ttc	ttTc	F	FX	0.0080
*TAS2R5*	rs2234014	Ns	cCg	cTg	P	L	0.0080
*TAS2R8*	rs41324347	Sg	Gaa	Taa	E	Stop	0.0076
*TAS2R1*	rs2234232	Ns	tGt	tAt	C	Y	0.0076
*TAS2R10*	rs201689842	Fs	Att	tt	I	X	0.0062
*TAS2R14*	rs35804287	Ns	Ctc	Ttc	L	F	0.0060
*TAS2R7*	rs150192473	Sg	Cga	Tga	R	Stop	0.0058
*TAS2R39*	rs184819681	Ns	aGc	aAc	S	N	0.0058
*TAS2R20*	rs116400924	Sg	tGg	tAg	W	Stop	0.0054
*TAS2R8*	rs142540719	Ns	aGa	aTa	R	I	0.0054
*TAS2R42*	rs139960283	Ns	tTg	tGg	L	W	0.0052
*TAS2R7*	rs139604652	Ns	gTg	gAg	V	E	0.0046
*TAS2R46*	rs150894148	Sg	Cag	Tag	Q	Stop	0.0042
*TAS2R13*	rs34885344	Ns	cAc	cGc	H	R	0.0042
*TAS2R41*	rs75955374	Ns	gTt	gAt	V	D	0.0040
*TAS2R19*	rs77837442	Sg	tgG	tgA	W	Stop	0.0038
*TAS2R10*	rs142507813	Ns	atG	atA	M	I	0.0038
*TAS2R10*	rs114006371	Ns	aTc	aAc	I	N	0.0038
*TAS2R19*	rs115193179	Ns	aAg	aCg	K	T	0.0038
*TAS2R40*	rs565742335	If	gAGAag	gag	EK	E	0.0036
*TAS2R7*	rs202246571	Ns	cTc	cAc	L	H	0.0034
*TAS2R31*	rs140958087	Ns	gGa	gTa	G	V	0.0034
*TAS2R5*	rs2234013	Ns	Ggt	Agt	G	S	0.0030
*TAS2R8*	rs61737282	Ns	aTa	aCa	I	T	0.0026
*TAS2R9*	rs148917754	Ns	tCa	tTa	S	L	0.0022
*TAS2R8*	rs114977408	Ns	Tac	Cac	Y	H	0.0020
*TAS2R31*	rs202165116	Ns	tTa	tCa	L	S	0.0020
*TAS2R38*	rs115966953	Ns	cGg	cAg	R	Q	0.0020

Nucleotide diversity ranged from 0.005% in *TAS2R39* to 0.358% in *TAS2R20*, with a mean of 0.12%. Comparing the observed π values in *TAS2R*s with the genome wide empirical distribution revealed that 20 of the 23 of observed values fell between the 5th and 95th percentiles, making them consistent with neutral expectations (Table 3). Two genes, *TAS2R20* and *TAS2R42*, had π values above the 95th percentile (percentiles 98.6 and 98.2 respectively). One, *TAS2R39*, had a π value below the 5th percentile, 0.005%. The mean π, 0.0012 fell at the 72nd percentile of the genomic empirical distribution, again well within expectations.

Of the 721 SNPS, 525 were nonsynonymous substitutions and 196 were synonymous. Among these, PolyPhen-2 and SIFT detected 239 variants predicted to alter TAS2R receptor function. Overall, 182 SNPs had PolyPhen-2 scores of Possibly or Probably Damaging and 188 had SIFT scores of Deleterious. The prediction tools were largely in agreement, with 131 SNPs predicted to be Possibly or Probably Damaging by PolyPhen-2 and Deleterious by SIFT. SIFT and PolyPhen-2 scores were also in agreement for 258 nonsynonymous SNPs predicted to be Benign by PolyPhen-2 and Tolerated by SIFT. Among sites where classification disagreed between the two methods, 51 were scored by PolyPhen-2 to be Possibly or Probably Damaging but not Deleterious by SIFT, and 57 were scored by were SIFT to be Deleterious but not Possibly or Probably damaging by PolyPhen-2. Sites likely to have functional effects but not scored by SIFT or PolyPhen-2 because they are not amino acid changes were also present. They included 27 SNPs resulting in premature stop codons, one resulting in the loss of a stop codon, and three resulting in a lost start codon. There were also nine small indels, seven of which resulted in frameshifts.

Because their characteristics predict that they will have functional effects, we denoted variants scored as possibly or probably damaging by PolyPhen and Deleterious by SIFT, indels, and changes altering start or stop codons as putatively high impact (PHI) sites, and their derived alleles PHI alleles. Under these criteria, we categorized 131 nonsynonymous SNPs along with the 9 indels as (PHI) sites. Notably, every *TAS2R* harbored at least one PHI site. Like the overall distribution of allele frequencies, the distribution of allele frequencies at PHI sites was dominated by rare variants. Only two PHI alleles had frequencies above 0.20 and eight had frequencies above 0.01. The mean derived allele frequency among PHI sites was 0.004, with a minimum of 0.0002 and a maximum of 0.22.

**FIGURE 1:**
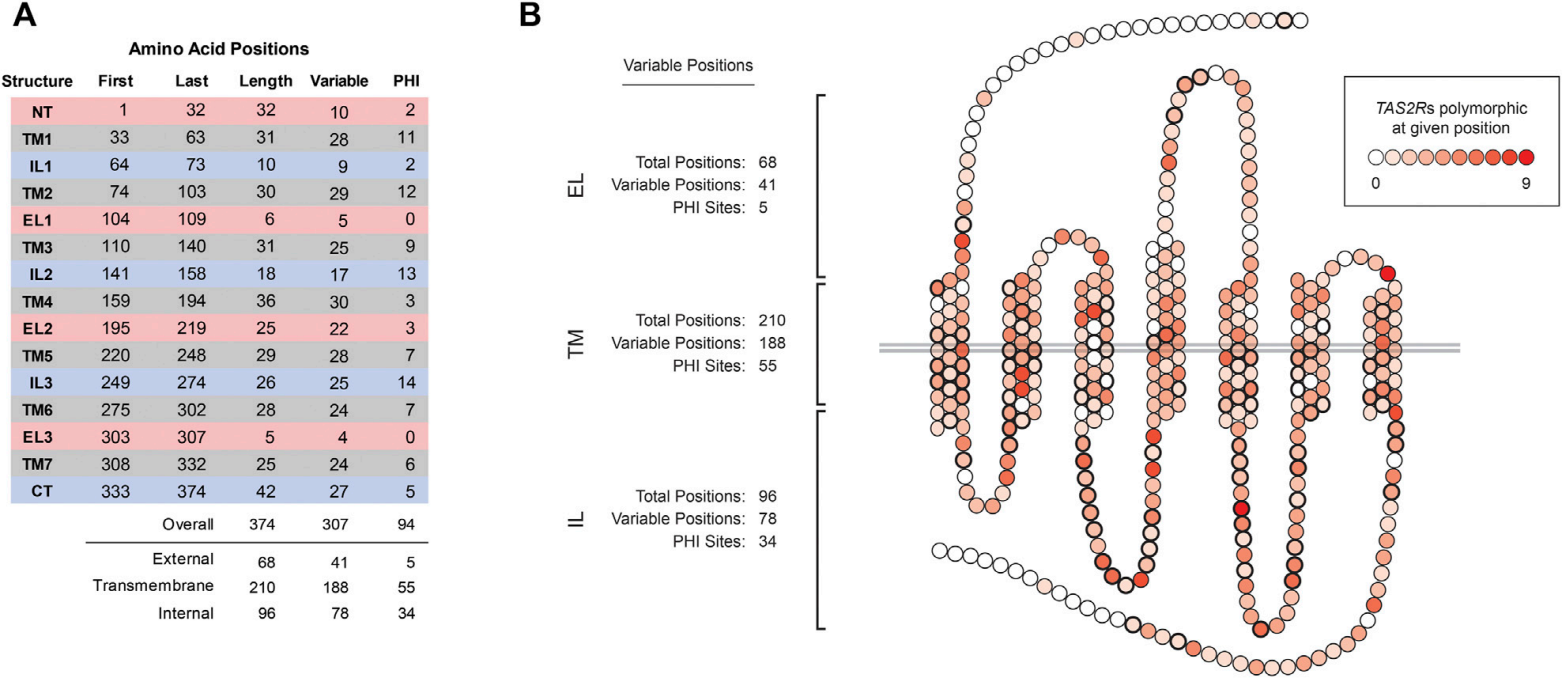
Variation across TAS2R substructures. **(A)** Coordinates, lengths, and numbers of variable and PHI sites in external loops (ELs), transmembrane (TMs), and internal loops (ILs). N-terminus and C-terminus sites were categorized as EL and IL, respectively. **(B)** Distribution of variability across amino acid positions. Shading indicates the number of TAS2Rs variable at the indicated position, which ranged from 0 to 9 of 23 receptors. Positions affected by PHI variants are indicated in bold.

Comparisons of the aligned TAS2R sequences revealed that polymorphism was present at 307 of 374 sites (82%; Figure 1A). Variation was found in all regions of the molecule and PHI sites were found in all but two regions, EL1 and EL3, which are both short (6 and 5 residues, respectively). The number of compared TAS2Rs harboring polymorphism at a given position also varied (Figure 1B). Sixty-seven positions were invariant in all TAS2Rs. However, the majority of positions (205 of 374) were polymorphic in two or more TAS2Rs and one, position 307, was polymorphic in nine of the 23 TAS2Rs.

**FIGURE 2:**
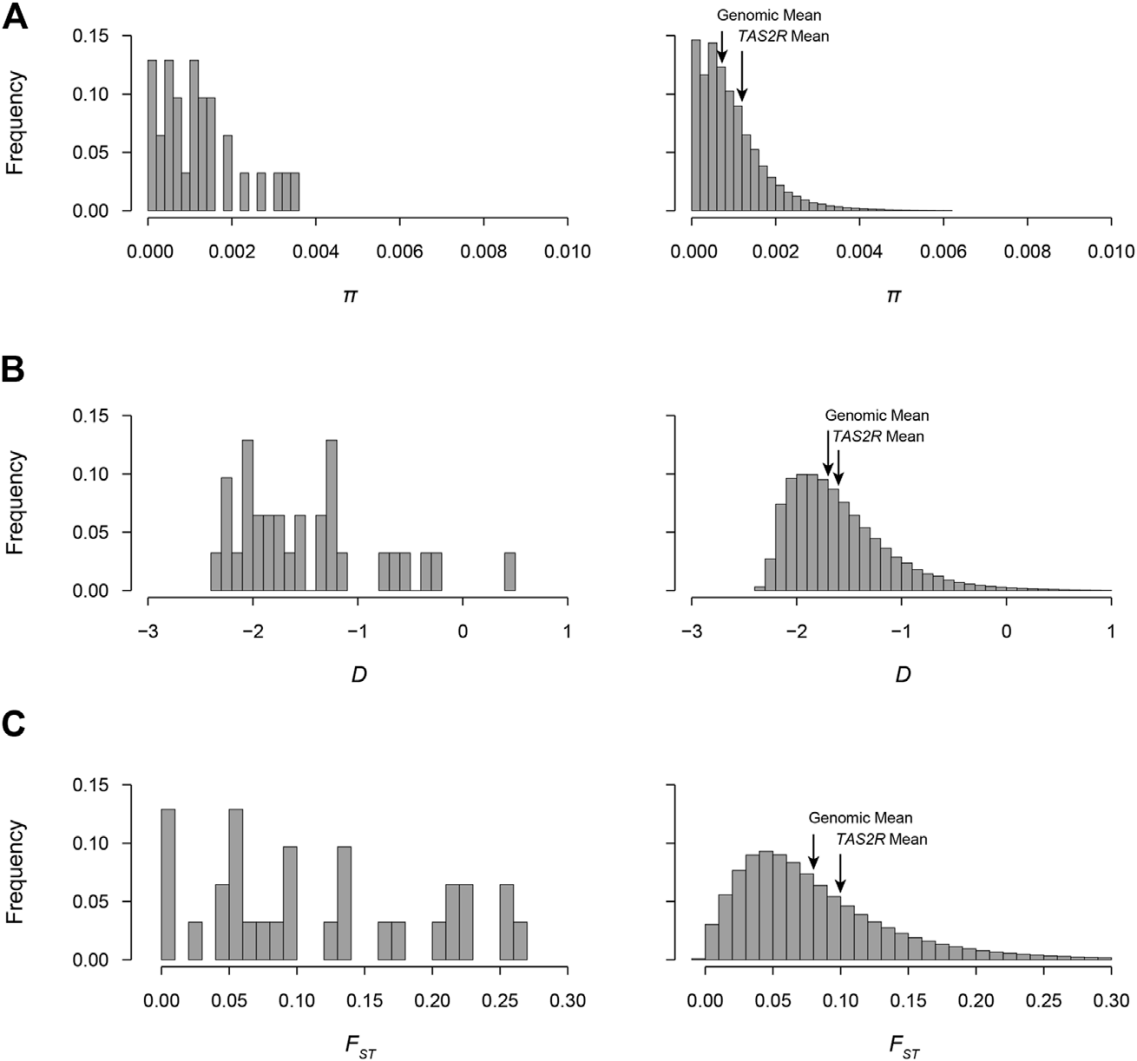
Distributions of π, Tajima’s *D*, and *F*_ST_ in the 1000GP. Values observed in *TAS2R*s and genome-wide sliding windows are shown in the left and right of each panel, respectively. **(A)** *π* distributions. Mean values in *TAS2R*s and genome-wide were 0.0012 and 0.0009, respectively. **(B)** *D* distributions. Mean values in *TAS2R*s and genome-wide were −1.61 and −1.59 and. **(C)** *F*_ST_ distributions. Mean values in *TAS2R*s and genome-wide were 0.13 and 0.08.

Tajima’s *D*, like the other diversity measures, varied substantially across loci. The highest and lowest values were observed at *TAS2R39* (*D* = −2.26; *P*_E_ = 0.055) and *TAS2R20* (*D* = −0.25; *P*_E_ = 0.98) and exceeded expectation under neutrality for *TAS2R20* and *TAS2R42* (*D* = −0.47; *P*_E_ = 0.97) (Figure 2). The mean *D* across loci was −1.61 (*P*_E_ = 0.60), which is consistent with the overall excess of low frequency variants across bitter receptor genes.

*F*_ST_ values ranged from 0.01 at *TAS2R39* (*P*_E_ = 0.026) to 0.258 at *TAS2R20* (*P*_E_ = 0.98). Differentiation exceeded expectation (*P*_E_ > 0.95) for *TAS2R8, 13, 16, 20, 42*, and 50 when compared to the empirical distribution, and fell below expectation for one, *TAS2R39* (*P*_E_ = 0.026) (Figure 2). The mean *F*_ST_ across loci was 0.13, which was consistent with previously published genome wide averages and lay at the 82nd percentile of the genomic distribution in the 1000GP (Bamshad and Wooding, 2003). *F*_ST_s for nonsynonymous sites ranged from 0.0009 at *TAS2R3* to 0.29 at *TAS2R50*. Among synonymous sites *F*_ST_ ranged from 0.00 in *TAS2R13*, which had a single rare substitution, to 0.30 in *TAS2R20*.

**FIGURE 3:**
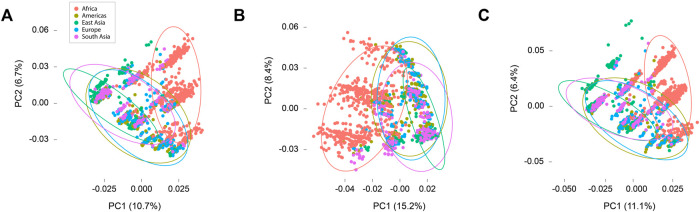
Principal components plots. **(A)** All sites. **(B)** Synonymous sites only. **(C)** Nonsynonymous sites only.

Principal components analyses revealed overlaps in diversity among super populations (Figure 3). In the combined analysis of synonymous and nonsynonymous sites the first and second principal components together accounted for 17.4% of observed variance. Analyses in synonymous and nonsynonymous sites separately yielded similar results, with the first two components accounting for 23.6% and 17.5% respectively. The results illustrated the extent of similarity and difference among individuals specifically with respect to variation in TAS2Rs, with coordinates near each other reflecting greater similarity and distant points reflecting less similarity. In all three data subsets (all sites, synonymous sites, and nonsynonymous sites), subjects from Asia, Europe, and the Americas showed extensive overlap, reflecting overlap in the variability of TAS2R loci. The African subjects were somewhat distinct, falling in an partially overlapping but somewhat different cluster in all analyses.

## Discussion

The broad importance of bitter perception to vertebrate biology raises questions about its specific importance to humans, who pose a conundrum. On the one hand humans have sophisticated cognitive abilities and might use cues other than bitter taste, such as learned recognition or social behavior, to gauge food safety. This could reduce selective pressures on *TAS2R*s. On the other hand, human history over last 65,000 years has been characterized by explosive population growth and rapid diffusion, which brought humans into contact with myriad novel environments (Bergström et al., 2021). This could have enhanced *TAS2R*s’ value for evaluating the toxicity of unfamiliar plants, increasing selective pressures on them. Moreover, these alternatives are not mutually exclusive, and might vary across loci. Our findings clarify global patterns of diversity in *TAS2R*s and evidence for selective pressures on them.

Consistent with previous studies, the variation we found across *TAS2R*s in the 1,000GP was extensive with respect to both synonymous and nonsynonymous polymorphism (Tables 3, 4). The mean number of segregating sites per locus in our sample (31.7 per locus), as well as the aggregate number across loci (721 SNPs) far exceeded all previous reports in family wide studies of *TAS2R*s. For instance, in a study across the *TAS2R* family in 22 globally distributed subjects Wang et al. (2004) observed 105 segregating sites (4.2 per locus), and a study in 55 subjects by Kim et al. (2005) yielded 151 sites (5.6 per locus). We observed a similar trend in single locus studies. While we cataloged 32 segregating sites at *TAS2R38* in the 1,000GP, Wooding et al. (2004) observed 5 in a sample of 155 global subjects and Campbell et al. (2012) observed 21 in a sample of 611 Africans. Similarly, we found 36 segregating sites at *TA2R16* whereas Campbell et al. (2014) found 15 across 689 worldwide subjects and Soranzo et al. (2005) found 17 across 997. Therefore, from an absolute standpoint our findings provide the largest and most comprehensive inventory of *TAS2R* variation to date.

A second feature of our data, an abundance of nonsynonymous variation, was also consistent with previous studies. We found that the mean number of segregating sites per locus in our sample, 31.7, was accounted for by a mean of 23.1 nonsynonymous and 8.6 synonymous sites, such that 73% of substitutions were nonsynonymous changes. This places our estimate intermediate between those reported in previous multi-locus studies such as the 69% rate reported Wang et al. (2004) and the 75% rate reported by Kim et al. (2005). Nonsynonymous rates were also similar for individual loci. For instance we observed a rate of 69% at *TAS2R38* while Campbell et al. (2012) found a 90% rate and Wooding et al. (2004) found a 100% rate. Likewise, we found a 56% rate at *TAS2R16* while Campbell et al. (2014) found a rate of 47% and Soranzo et al. (2005) found a rate of 64%. Across all loci in our study we found a substantial range of rates, from a minimum of 56% (at *TAS2R16*) to 94% (at *TAS2R13*), with a mean of 73%. Because amino acid substitutions can alter the properties of proteins, and hence their function, the abundant nonsynonymous variation in *TAS2R*s suggests that they could produce substantial variation in taste phenotypes.

While the presence of nonsynonymous variation points to the presence of functional variation, it does not demonstrate it. Some amino acid substitutions are more impactful than others as the result of chemical similarities between residues, their positions within the protein structure, and other factors. As a result, some substitutions have no discernable effects, while others have intermediate or strong ones. This has implications for the effects of substitutions on taste phenotypes. In particular, is likely that some of the substitutions we observed affect taste responses while others do not. These alternatives are exemplified in TAS2Rs by the findings of Roudnitzky et al. (2011). In a combined *in vitro* and psychophysical analysis of variation in TAS2R31, Roudnitzky et al. identified nine amino acid substitutions. Among these one, TAS2R31-R35W had strong effects on receptor function and was associated with taste responses to the bitter off-tastes of saccharin and acesulfame potassium. However, other substitutions had more complex effects. For instance, a TAS2R31-D45H substitution altered receptor function only when combined with the W35 allele. Amino acid replacements in other TAS2Rs exhibit graded effects. For instance, Roudnitzky et al. (2015) found that two amino acid replacements in TAS2R46 combine to form three alleles with high, medium, and low responses *in vitro*. Similarly, Wooding et al. found that amino acid substitutions in TAS2R38 differentially affect its functional responses and taste responses to phenylthiocarbamide (PTC) and a bitter compound found in vegetables, goitrin.

The computational predictions we obtained using SIFT and PolyPhen 2 were consistent with the suggestion that *TAS2R*s harbor extensive functional variation. In total, 131 SNPs had both high SIFT and high PolyPhen-2 scores, and were thus PHI sites, with an additional 35 PHI sites being altered stop codons, start codons and indels. Taken together, these findings point to the presence of numerous TAS2R variants capable of affecting taste phenotypes. Nonetheless, the frequency distribution of PHI alleles suggested that the contributions of most sites to phenotypic variance are small (Table 4). The distribution was strongly skewed downward such that although there were 166 PHI sites, just 21 had frequencies above 0.005 and eight had frequencies above 0.01. Only two sites were found at moderate frequencies, a premature stop in *TAS2R46* (rs2708381) with a frequency of 0.21 and an arginine-glutamine substitution in *TAS2R42* (rs1669412) with a frequency of 0.22. In addition, while most individual sites and all subdomains of TAS2Rs harbored variation, it was not obviously concentrated in any region of the molecule. Thus, while scores of variants in *TAS2R*s were computationally predicted to result in functional variation, most of them were rare so their effects must be limited to a small number of subjects and contribute little to phenotypic variance more broadly.

Cross referencing PHI sites against functional sites detected in previous studies revealed potential weaknesses in the ability of SIFT and PolyPhen-2 to identify variants with phenotypic effects. In particular, the PHI sites identified with SIFT and PolyPhen-2 showed no overlap with sites previously demonstrated to have functional and phenotypic consequences. These included three sites in *TAS2R38* known to mediate taste responses to PTC and PROP (Bufe et al., 2005), two sites in *TAS2R43* and -*44* mediating responses to aloin, saccharin, and acesulfame potassium (Pronin et al., 2007; Roudnitzky et al., 2011), and two sites in *TAS2R16* mediating taste responses to glucopyranosides (Soranzo et al., 2005). Importantly, the lack of overlap between PHI sites and known functional sites does not imply a failure on the part of SIFT and PolyPhen-2. First, the number of confirmed functional, phenotypically important polymorphisms in human *TAS2R*s is currently small, limiting the potential for overlap. Second, both algorithms have high true positive rates and hence also have high false negative rates. This might have resulted in a high type-2 error rate in our analyses. Further, these algorithms are aimed at detecting the effects of changes at individual sites, not the effects of site-site interactions, which are known to occur in TAS2Rs, such as the aforementioned interactions at TAS2R31. Nonetheless, given that functional variation in TAS2Rs has been demonstrated unambiguously *in vitro*, we hypothesize that *TAS2R*s do harbor substantial functional variation. They may be elucidated more effectively recent and sophisticated methods such as computational inferences, which are increasingly powerful (Bahia et al., 2018; Dagan-Wiener et al., 2019; Margulis et al., 2021). The emergence of new techniques for determining proteins’ three dimensional structures, such as AlphaFold may be a particularly effective resource (Jumper et al., 2021).

Natural selection has long been recognized as a probable influence on bitter taste perception (Fisher et al., 1939). Because it is directly involved in feeding behaviors bitter perception seems likely to affect reproductive success, placing it under selective pressure. However, previous studies of natural selection in human *TAS2R*s have reached contrasting conclusions. For instance, while Wooding et al. (2004) found patterns of diversity consistent with the effects of natural selection on *TAS2R*s, Wang et al. (2004) concluded that selective pressures on *TAS2R*s are relaxed. Our findings in the 1,000GP are more consistent with the latter perspective. Among the 23 loci we examined, only two had *D* values above the 95th percentile in the empirical distribution, which was assumed to represent neutral expectations (*TAS2R20*, *P*_E_ = 0.98; *TAS2R42*, *P*_E_ = 0.97) (Table 3). These two measures reflect significantly high *D* values, which typically result from diversifying pressures such as balancing natural selection or local adaptation {Enard, 2021 #1976}. Thus, while some loci with evidence of selection were present, more than 90% had *D* values consistent with selective neutrality. Further, the mean *D* value in *TAS2R*s was also close to the mean of the genome wide empirical distribution (−1.61 vs. −1.59, *P*_E_ = 0.57), the result expected under neutrality (Figure 2). Overall, this is evidence for a general decrease or absence of selective pressure on *TAS2R*s in modern human populations, but there are important exceptions that merit further investigation.

Evidence that selective pressures have been relaxed on *TAS2R*s raises questions about the timing of relaxation. One possibility is that selective pressures on *TAS2R*s shifted with the advent of domestication, which enabled humans to produce large quantities of food using nontoxic crops such as wheat, rice, and corn. However, plants were first domesticated just ∼10,000 years ago (Gross and Olsen, 2010). It is conceivable that the equilibration of diversity in *TAS2R*s from expectations under selection to neutral expectations occurred in that short time frame, but it may have taken place over a more extended period. We speculate that the relaxation of selective pressures on *TAS2R*s is better explained by longer term factors in human evolution and behavior, particularly social behavior, learned foraging strategies, and cognitive developments. These have likely enhanced humans’ ability to forage safely and efficiently without cues from taste for hundreds of thousands of years. If so, *TAS2R*s probably began evolving neutrally prior to humans’ dispersal from Africa. This would predict that other genes implicated in coping with phytotoxins, such as cytochrome P40 genes, experienced shifts in selective pressures over the time period. It would also predict that most or all *TAS2R*s experienced reductions in selective pressure simultaneously. These suggestions are potentially testable using phylogenetic methods to compare TAS2Rs in humans with those in other primates or with genes predicted to be under different selective pressures over the same time period.

The proposal that human populations differ with respect to taste sensitivity emerged as soon as phenotypic variation in taste responses was discovered (Fox, 1932). Our findings suggest populations do differ with respect to functional variation at some *TAS2R* loci. However, the differences overall were no greater than expected given the genome wide neutral distribution. First, *F*_ST_ under panmixia, in which there is no differentiation among populations, is 0. We found that only one gene, *TAS2R39*, had an *F*_ST_ with *P*_E_ < 0.05, and was thus distributed more homogeneously than expected by chance. The remaining 22 *TAS2R*s had *F*_ST_s with *P*_E_ > 0.05, which is consistent with the presence of at least some genetic differentiation. In addition, six loci (*TAS2R8*, -*13*, -*16*, -*20*, -*42*, and -*50*) had *P*_E_ values > 0.95, and thus showed strong evidence of excess genetic differentiation that could result in phenotypic differences. Moreover, the *F*_ST_ at *TAS2R13*, 0.23 (*P*_E_ = 0.97), was accounted for entirely by nonsynonymous variation, suggesting that it is particularly likely to underlie population differences. However, while a number of individual loci showed evidence of departing from expectations, the distribution of *F*_ST_ across the *TAS2R* family as a whole did not reveal differences from the genome side empirical distribution. When compared across loci, the mean *F*_ST_ observed across *TAS2R*s, 0.13, was somewhat higher than the genome wide mean but not extremely so, falling in the 81.5th percentile of the overall distribution (Figure 2). This suggests that populations are differentiated with respect to variation in *TAS2R*s, but most loci are no more differentiated than expected under neutrality.

The extent of overlap among populations with respect to variation in *TAS2R*s was emphasized by the results of principal components analyses (Figure 3). These revealed that the African super population was somewhat distinct from the others in every analysis, falling in an overlapping but slightly separate cluster from the other four super populations. This is a common pattern in humans (Bamshad et al., 2004). Nonetheless, the pattern in nonsynonymous variants was noteworthy. While SIFT and PolyPhen-2 did not detect high frequency functional variants, their high false negative rates suggest that many functional variants could be present but undetected. Therefore, while the results of principal components analyses point to overall similarities in population differentiation with respect to synonymous and nonsynonymous variation, the presence of differentiation with respect to nonsynonymous sites suggests that there could be systematic differences among super populations with respect to those sites and, hence, with respect to their respective bitter taste phenotypes. Differences between African and non-African populations are particularly likely.

Variation in bitter taste sensitivity has long been recognized as a potential health factor because it is tied to ingestive behaviors, many of which have potential consequences for health. For instance, connections between taste sensitivity to PTC and thyroid health were proposed as early as 1949 (Harris et al., 1949). Later studies proposed connections between bitter taste and food preferences more generally, and between bitter taste and tobacco use (Fox, 1954; Fischer et al., 1963). The emergence of molecular genetic technologies transformed perspectives on connections between bitter taste perception and health by uncovering many specific compounds eliciting bitter responses as well as the *TAS2R*s controlling them. The most complete portrait of these relationships is exemplified by *TAS2R38*. *TAS2R38* is responsive to a range of isothiocyanates (ITCs) impairing thyroid activity and harbors variants altering TAS2R38’s responses to them. Further, *TAS2R38* genotypes associate with taste responses to ITCs and preferences for foods containing them, as well as with downstream health traits shaped by ingestion behaviors, such as body mass index (Ortega et al., 2016).

Relationships between genetic variation, taste responses and health involving *TAS2R* loci other than *TAS2R38* are not as well defined, but they show evidence of similar effects. For instance the association of taste sensitivity to the bitter off-taste of noncaloric sweeteners with variants in *TAS2R31* suggests they may influence preferences for diet foods, with possible consequences for weight loss and long term BMI (Roudnitzky et al., 2011). Variants in *TAS2R43* affect the receptor’s responses to aristolochic acid, a carcinogenic contaminant of wheat supplies in Eastern Europe, and predict kidney damage in exposed populations (Grollman et al., 2007; Pronin et al., 2007; Wooding et al., 2012). Other potentially important associations include relationships between variants in *TAS2R16*, receptor response, and the perception of salicin, and between variants in *TAS2R3*, -*4*, and -*5* and perception of ethanol, suggesting they may shape consumption and its health effects (Campbell et al., 2014; Nolden et al., 2016). Our finding that *TAS2R*s harbor hundreds of nonsynonymous variants suggests that the responses they mediate may be similarly extensive and can be anticipated to emerge in future studies.

It is important to note that while much focus in research on bitter taste and health has been on individual TAS2Rs, it is well recognized that other factors are important, particularly when considering complex phenotypes such as food and drink likings (Dioszegi et al., 2019). These include the potential for contributions from multiple TAS2Rs simultaneously, contributions from individual TAS2Rs to multiple traits, contributions from other molecules participating in taste such as salivary proteins, contributions from neurological systems, and environmental factors. An important development in dissecting these has been the emergence of extremely large population samples, some of which now exceed 100,000 subjects. These provide a statistically powerful means of detecting both predicted and unpredicted influences. For instance, a recent study of the UK-Biobank (> 160,000 subjects) by May-Wilson et al. (2022) detected more than a thousand associations with food and drink likings, which implicated not just TAS2Rs but myriad other genes as well, such as FGF21 (fibroblast growth factor), ADH1B (alcohol dehydrogenase), and MHC (major histocompatibility complex) loci.

While *TAS2R*s are best known for their roles mediating bitter taste responses and ingestive behaviors, mounting evidence indicates that they play important roles outside perception, as well. For instance, they are highly expressed in cells in gut and bronchial smooth muscle responsive to ingested and inhaled compounds (Calvo and Egan, 2015; Cohen, 2017; An and Liggett, 2018; Tran et al., 2021). For instance, stimulation of gut expressed *TAS2R*s triggers endocrine responses and gastric emptying (Dotson et al., 2008; Egan and Margolskee, 2008). Evidence that TAS2Rs can monitor quorum sensing in intestinal flora raises the possibility that they also mediate responses to changing conditions in the gut (Cohen, 2017; Fan and Pedersen, 2021). Recent findings point to another function of TAS2Rs in the gut, the detection of parasites emitting TAS2R agonists (Luo et al., 2019). Similar patterns are found in the airways, where TAS2Rs trigger responses to both endogenous and exogenous compounds entering the lungs, and likely mediate immune responses (Cohen, 2017; Tran et al., 2021). Investigations of the impact of genetic variation in *TAS2R*s on these processes are so far scant, but those available point to important effects. For example, consistent with evidence that ingestion of bitter compounds can produce hormonal responses in gut cells, Dotson et al. (Dotson et al., 2008) found that variation in *TAS2R9* associates with glucose dysregulation and Clark et al. (Clark et al., 2015) found that variation in *TAS2R42* associates with thyroid hormone levels. Similarly, in an investigation of variation of associations between TAS2R38 variants and respiratory health, Lee et al. (Lee et al., 2012) found that variation in *TAS2R38* associates with susceptibility to infection.

## Conclusion

Our findings highlight the complexity of relationships between bitter taste receptors, perception, evolution, and health. From a broad perspective, the patterns we observe across the *TAS2R* family point to a reduction in selective pressure in the course of human evolution, possibly as the result of evolved changes in other aspects of human biology and behavior. However, some loci do show evidence of departures from neutrality that may represent recent or ongoing influences from selection. Further, while computational analyses in our sample did not identify high frequency sites predicted to have functional effects, they did not exclude them and the presence of hundreds of nonsynonymous variants, including some with high frequencies and frequency differences among populations, raises the possibility that they shape TAS2R mediated phenotypes. Ongoing developments in molecular genetics and computational biology make dissecting their effects a compelling prospect.

## Data Availability

Publicly available datasets were analyzed in this study. This data can be found here: 1000 Genomes Project (https://www.internationalgenome.org/). Data files used for analysis are provided in the article Supplementary Material.
